# Seroconversion dynamic and SARS-CoV-2 seropositivity in unvaccinated population during the first and second outbreaks in Mexico

**DOI:** 10.1038/s41598-022-09395-3

**Published:** 2022-03-28

**Authors:** Miguel A. Fernández-Rojas, Marco A. Luna-Ruiz Esparza, Abraham Campos-Romero, Diana Y. Calva-Espinosa, José L. Moreno-Camacho, Fela Mendlovic, Tanya Plett-Torres, Jonathan Alcántar-Fernández

**Affiliations:** 1 Innovation and Research Department, Salud Digna A.C., Francisco Villa 113 sur, 80000 Culiacán, Sinaloa Mexico; 2 Clinical Laboratory Department, Salud Digna, 80000 Culiacán, Sinaloa Mexico; 3 National Reference Center “Culiacan”, Salud Digna, 80300 Culiacán, Sinaloa Mexico; 4 Departamento de Microbiología y Parasitología, Facultad de Medicina, Universidad Nacional Autónoma de México, Mexico City, Mexico; 5 Facultad de Ciencias de la Salud, Universidad Anáhuac México Norte, Huixquilucan, Estado de Mexico Mexico; 6 Plan de Estudios Combinados en Medicina, Facultad de Medicina, Universidad Nacional Autónoma de México, Mexico City, Mexico

**Keywords:** Epidemiology, Epidemiology

## Abstract

Serosurveillance helps establish reopening guidelines and determine the immunity levels in different populations to reach herd immunity. Then, there is an urgent need to estimate seroprevalence population wide. In Mexico, information about COVID-19 cases and related deaths is scarce. Also, there is no official serosurveillance, limiting our knowledge of the impact of the SARS-CoV-2 pandemic. Here, we report the prevalence of anti-SARS-CoV-2 antibodies in 522,690 unvaccinated people from July 5th to December 31st, 2020. The overall seroprevalence was 32.8% and highest in adults aged 30–39 years (38.5%) than people under 20 years (33.0%) or older (28.9%). Moreover, in a cohort of 1655 individuals confirmed COVID-19 by PCR, we found that symptomatic people (HR = 2.56) increased seroconversion than presymptomatic. Also, we identified that the most discriminative symptoms for COVID-19 that could predict seroconversion were anosmia and ageusia (HR = 1.70), fever, myalgia/arthralgia, and cough (HR = 1.75). Finally, we found that obese people had lower seroconversion (HR = 0.53) than healthy people, but the opposite happens in diabetic people (HR = 1.39). These findings reveal that around one-third of Mexican outpatients had anti-SARS-CoV-2 antibodies before vaccination. Also, some symptoms improve empirically COVID-19 diagnosis and seroconversion. This information could help fine-tune vaccination schemes and the reopening and back-to-work algorithms.

## Introduction

Epidemiological serosurveillance could be helpful to understand the exposure levels to the SARS-CoV-2 infection and make inferences on the burden of COVID-19 at the geographical level and specific population groups. Some studies have shown that immunological tests could also help detect positive cases in asymptomatic people^1,2^. Thus, although these tests do not help recognize an acute infection, they could help detect asymptomatic people since antibody tests can detect the disease after 5–7 days^3^. Also, most reports come from hospitalized people^4^ and few from community settings, outpatients, asymptomatic or presymptomatic people^5,6^. Therefore, the study of the natural immunity acquired after SARS-CoV-2 infection and its related factors is of interest to estimate the effectiveness of immunity mediated through vaccination. That information could help policymakers refine the extent of the ongoing vaccination programs and make interventions to prevent outbreaks, design back-to-work algorithms, and control the pandemic^7^. More studies are needed to understand the immunity response against SARS-CoV-2 better.

In Mexico, the first positive case of SARS-CoV-2 infection was reported on February 27th, 2020. To this date, 5,436,566 confirmed cases and 316,492 deaths associated with COVID-19 had been documented^8^. Moreover, in its last analysis, the Mexican National Institute of Statistic and Geography (INEGI) pointed out that positive cases and deaths are underestimated, implying the lack of reliable data about the spread of SARS-CoV-2 in the country^9^.

Moreover, previous reports of serosurveillance were restricted to some states, public health institutions, or high-risk populations like healthcare workers^10–13^. Therefore, this study aimed to analyze information collected during the first and second SARS-CoV-2 epidemic outbreak before vaccination to understand the pandemic impact in Mexico and identify clinical factors associated with natural seroconversion. To this, we analyze information belong people tested for antibodies against SARS-CoV-2 in outpatient diagnostic Salud Digna clinics (from the private medical sector in Mexico) in 2020 during the first and second viral wave in the country before vaccination.

## Methods

### Study design and population studied

We retrospectively analyze anonymized electronic health records from 522,690 unvaccinated outpatients tested for SARS-CoV-2 antibodies at the Salud Digna Clinics throughout Mexico from July 5th to December 31st, 2020.

### Data collection and consent for using information

According to the Mexican Federal Law on Personal Data Protection (LFPDPPP, by its acronyms in Spanish), consent for the use of information from health records was obtained from each individual included in this work as follows. People receiving diagnostic and health care services in the Salud Digna clinics accept our privacy policy, which includes using their anonymized information for scientific research purposes. Also, people can deny the use of their information at any time through the personal data protection department of Salud Digna in adherence to the Mexican federal law (LFPDPPP). By the above, we do not need specific, informed consent from each person included in this work because this study is a cross-sectional analysis of an electronic health registry.

### Handling data and protecting information privacy

According to Federal Law on Personal Data Protection in Mexico (LFPDPPP), we handle data protection and privacy. We assign a unique ID code to each registry that is not linked to the identity of individuals and to prevent data duplication; also, information is aggregated to enhance the protection of the identity and privacy of individuals.

### Inclusion and exclusion criteria

Only people allowed to use their information, with serological results (positive or negative) and demographic information, were included in the analysis. The general seroprevalence and by states was done with the entire dataset (522,690 people). For the prevalence by state, previously, we determined the minimum number of people tested to represent a state (381 people) according to sample size calculation for cross-sectional studies^14^ using the national COVID-19 incidence (45%) reported for the same period of study by the Ministry of Health in Mexico^15^. We found that people tested in each state exceeded the threshold (381 people), so we included all country states (Supplementary Table 1).

### The cohort of seroconversion analysis

Seroconversion is defined as a positive serological test in individuals previously diagnosed with COVID-19 by a real-time RT-PCR test; to analyze factors associated with seropositivity and seroconversion dynamic, we made a cohort of seroconverted people. In these subsets of people, we defined cases as symptomatic or presymptomatic according to the presence or absence of symptoms at the PCR test. Due to lack of follow-up patients was not possible to determine if they were genuinely asymptomatic or then after laboratory diagnostic, they have symptoms^16,17^.

In the entire electronic registry, we identify 14,592 people with a positive PCR test (≤ 14 days) previous to antibodies test; from this we assess the association of clinical and socioeconomic factors with seropositivity in this group. Also, from this subset (14,592 seroconverted people) we identify people (1655 individuals) with serial serological tests 180 days after the positive PCR test. Distribution of number of tests over time for each characteristic analyzed is showed in Supplementary Figs. S1-2.

For seroconversion dynamic, presymptomatic individuals were excluded to assess the impact of symptoms, and symptomatic people who did not experience the symptom analyzed were the reference group.

### Serological assay

Serological analyses were performed in peripheral blood samples collected from people attendant in Salud Digna Clinics and analyzed for anti-SARS-CoV-2 antibodies by electrochemiluminescence immunoassay (ECLIA) using qualitative Elecsys^®^ Anti-SARS-CoV-2 probe (Roche Diagnostics, US), which detects IgM and IgG antibodies and reports a sensitivity of 99.5% and a specificity of 99.8%. Sample processing and interpretation results (positive: COI ≥ 1 or negative: COI < 1) were according to manufacturer instructions using the module Cobas e602^®^ (Roche Diagnostics, US).

### Ethical approval

This study was approved by the Salud Digna Ethical Research and Review Committee (SDI-2020-2); The methods in this work adhered to the Helsinki declaration and were carried out according to the Federal law of Personal Data Protection in Mexico. Also, the committee evaluated and approved data collection and privacy protection procedures used in this study.

### Statistical analysis

We did a descriptive analysis for all variables in the dataset. Age groups were defined by decades, except for underrepresented groups (< 20 and > 60 years). The seroprevalence was obtained across the country, standardized by age and sex for every 100 persons, and calculated by the direct method using the standard world population as a reference; the confidence interval was obtained considering a Poisson distribution for ASR according to^18^.

Age and sex-adjusted or unadjusted multivariate logistic regressions were carried out as appropriate to evaluate the association between SARS-CoV-2 antibodies and demographic and clinical factors. A backward step-by-step analysis was used to select variables (p < 0.001) to adjust the final model. We assess the significance of demographic factors and symptoms for seropositivity with the Chi-square test.

Time-to-event analyses are shown by Kaplan–Meier curves from the time in which people tested positive by PCR test to a positive antibodies test result. Hazard ratios (HR) were calculated by Cox proportional hazards models, adjusted for sex and age (in decades), and time (number of days since PCR positive test) to account for the time from SARS-CoV-2 detection to seroconversion (positive results of antibodies test). We used the log-rank test (Mantel–Cox) to assess the significance of variables analyzed in Cox models.

We make analyses using SPSS23 (SPSS Inc., Chicago, Ill., USA) and time-to-event models using R 4.02 (CRAN project) and the survival (v3.1) package. Some graphs were made with GraphPad Prism 8 (GraphPad Software Inc., San Diego, CA.) and R 4.02 (CRAN project).

## Results

### Temporal trends and prevalence of seropositivity by state

**Figure 1:**
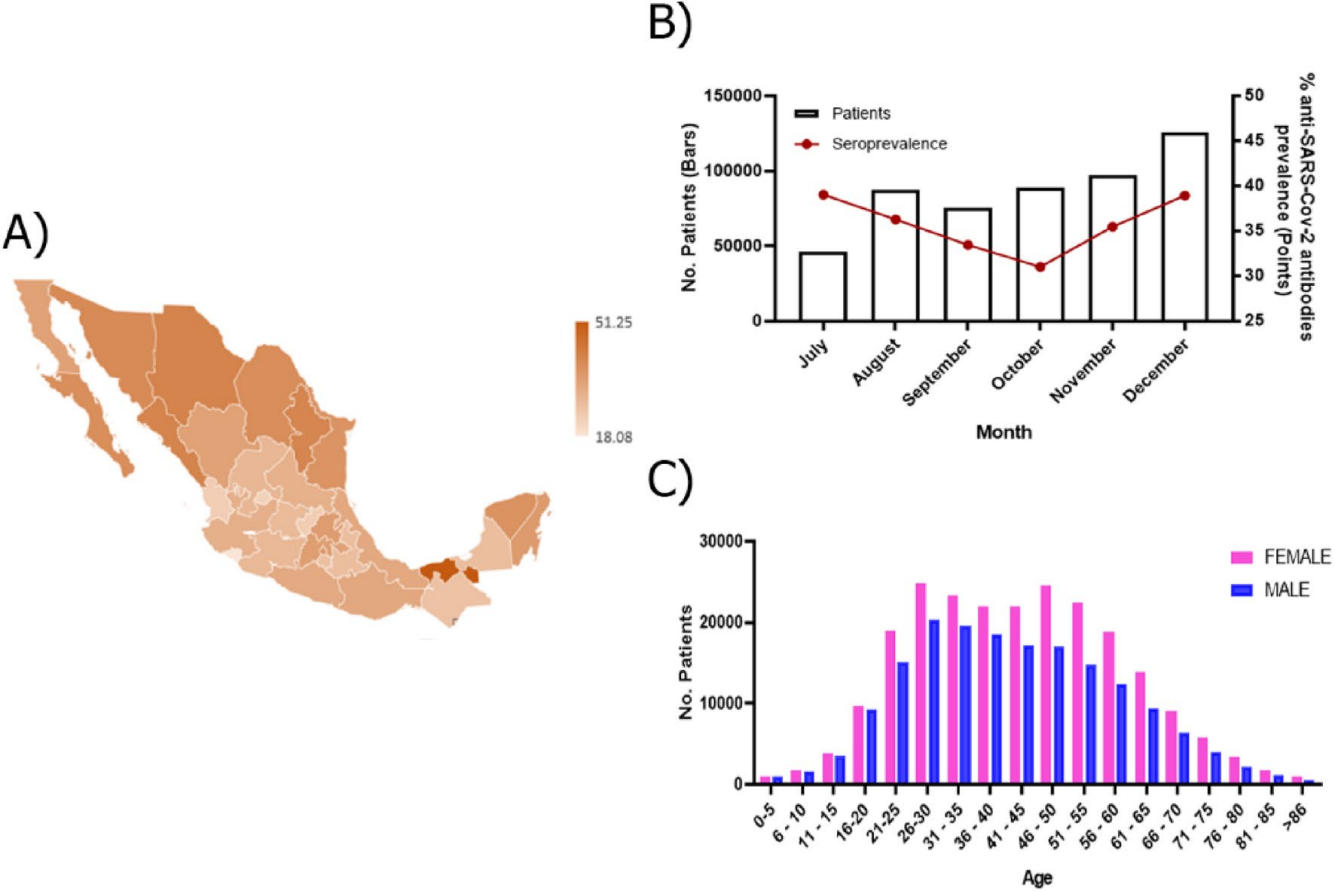
Overview of seroprevalence of SARS-CoV-2 in Mexico. (**A**) Coropletic map shows the anti-SARS-CoV-2 antibodies (IgM and/or IgG) prevalence in Mexico; as the prevalence increases, the brown color becomes darker; (**B**) Temporal trend and monthly seroprevalence to SARS-CoV-2 in 522,690 unvaccinated outpatients from July 5th to December 31st, 2020; (**C**) Distribution of seropositive SARS-CoV 2 cases according to age and sex in 522,690 unvaccinated outpatients.

This work includes information from all country states (32 states) from July 5th to December 31st, 2020. The total prevalence of anti-SARS-CoV-2 antibodies was 32.8% the lower prevalence was in Colima (18.1%), Aguascalientes (22.7%), Nayarit (23.7%), and Queretaro (23.9%), while in Tabasco (51.3%), Chihuahua (41.2%), Sinaloa (40.9%) and Nuevo León (40.7%) we found a higher prevalence. However, no specific geographic pattern was observed (Fig. 1A, Supplementary Table 1).

Moreover, the temporal analysis showed a reduction of antibodies' prevalence from July to October (39.0% to 31.0%, p < 0.0001); however, it increased again in December (38.9%, p < 0.0001) (Fig. 1B), which was consistent with the behavior of the first and second epidemic curves of SARS-CoV-2 pandemic in the country^8^.

### Characteristics of the study population

From July 5th to December 31st, 2020, 522,690 individuals were tested against SARS-CoV-2 antibodies, 32.8% (186,663 individuals; 95% CI 32.1–33.5, Supplementary Table 1) were positive, the median age of positive cases was 40 years (IQR: 29–52 years), and the 30–39 aged group had the highest prevalence of antibodies (Fig. 1C, Table 1). Also, we found that 56.0% of seropositive cases were in women (104,439 individuals) while 44.0% were men (82,224 individuals), however, age and sex standardized rate ratio did not show difference between sex (females = 32.6% (95% CI 31.6–33.7) vs males = 33.0% (95% CI 32.1–33.9), p = 0.584) (Fig. 1C, Table 1).

**Table 1:** Seroprevalence by age and sex of SARS-CoV-2 antibodies in 522,690 unvaccinated people.

Characteristic	Number of people n (%)	People positive to serological test	Prevalence of antibodies % (95% CI)	*p*-value
**Sex**				
Female	296,621	104,439 (56.0)	35.2 (35.0–35.4)	0.584
ASR female	–	–	32.6 (31.6–33.7)	
Male	226,069	82,224 (44.0)	36.4 (36.2–36.6)	
ASR male	–	–	33.0 (32.1–33.9)	
**Age (year)**^**a**^				
Median (IQR) = 40 (23)		–	–	–
< 20	34,797	11,474	33.0 (32.5–33.5)	< 0.0001
20–29	97,831	36,142	36.9 (36.6–37.3)	< 0.0001
30–39	109,828	42,330	38.5 (38.3–38.8)	Ref
40–49	104,108	39,536	38.0 (37.7–38.3)	0.0071
50–59	91,683	32,790	35.8 (35.5–36.1)	< 0.0001
≥ 60	84,443	24,391	28.9 (28.6–29.2)	< 0.0001

ASR = age and sex standardized rate per 100 inhabitants. IQR: interquartile range. ref = reference group.

^a^*p*-values for *x*^2^ test seroprevalence within groups.

### Characteristic of seroconverted people

We identified 14,592 individuals previously diagnosed with COVID-19 and tested by serological assays, of which 11,577 (79.3%) had antibodies against SARS-CoV-2.

Most of the screened patients had social security (81.0%) and did not receive the influenza vaccine in the last year (76.8%) (Table 2). Interestingly, we found a higher prevalence (83.4%) in cases without contact with confirmed cases of COVID-19 (Table 2). We identified that jobs like delivery people (OR = 2.2, 95% CI = 1.1–4.2, p = 0.019), logistic or transport workers (OR = 1.6, 95% CI = 1.2–2.2, p = 0.006) and informal traders (OR = 1.4, 95% CI = 1.1–1.8, p = 0.010), were related to anti-SARS-CoV-2 antibodies positivity (Supplementary Table 2).

**Table 2:** Clinical and lifestyle characteristics of 14,592 people with SARS-CoV-2 antibodies previously diagnosed with COVID-19 by PCR test.

Characteristic	Number of people n (%)	Prevalence of antibodies (%)	*p*-value*
**Comorbidity**			
No	12,562 (86.1)	9939 (79.1)	–
Yes	2030 (13.9)	1638 (80.7)	0.111
**Type of patient**			
Presymptomatic	630 (4.3)	341 (54.1)	**–**
Symptomatic	13,962 (95.7)	11,236 (80.5)	< 0.0001
**Contact with COVID-19 patient**			
No	6711 (46.0)	5597 (83.4)	**–**
Yes	7881 (54.0)	5980 (75.9)	< 0.0001
**Relationship COVID-19 patient**^**a**^			
Family	4746 (60.2)	3733 (78.7)	0.162
Co-worker	1935 (24.6)	1392 (71.9)	< 0.0001
Friend	943 (12.0)	689 (73.1)	< 0.0001
Other	558 (7.1)	404 (72.4)	< 0.0001
**Pregnant women**			
No	7895 (97.4)	6136 (77.7)	**–**
Yes	208 (2.6)	170 (81.7)	0.177
**Social security**^**b**^			
No	1959 (19.0)	1586 (81.0)	**–**
Yes	8334 (81.0)	6522 (78.3)	0.009
**Influenza vaccination**^e^			
No	11,158 (76.8)	8859 (79.4)	**–**
Yes	3366 (23.2)	2665 (79.2)	0.790

^a^It should be noted that some categories can be counted more than once; ^b^Missing values = 4299; ^c^Missing values = 68.

*p-values for *x*^2^ test seroprevalence within groups.

Regarding clinical characteristics, comorbidities were present in 13.9% of people diagnosed with COVID-19 (Table 2); the most prevalent illnesses were smoking (16.2%), hypertension (7.1%), diabetes (5.8%), and obesity (2.4%) (Supplementary Table 3). Also, we identified 4.3% of presymptomatic cases (630 individuals), while in symptomatic cases (95.7%), headache (60.9%), myalgia or arthralgia (53.1%), and sore throat (49.8%) were the most typical symptoms. Additionally, we observed that anosmia (1056/1108 = 95.3%), ageusia (770/808 = 95.3%) and fever (3694/4104 = 90.0%) were significantly more common in seropositive cases (Table 3).

**Table 3:** Prevalence of antibodies by symptoms in 14,592 people diagnosed with COVID-19 by PCR test.

Symptom or combination	Number of people n (%)^a^	Prevalence of antibodies (%)	*p-*value
Headache	8887 (60.9)	80.93	0.076
Myalgia or arthralgia	7745 (53.1)	84.70	** < 0.0001**
Sore throat	7272 (49.8)	82.77	** < 0.0001**
Cough	4847 (33.2)	89.33	** < 0.0001**
Runny nose	4605 (31.6)	85.54	** < 0.0001**
Fever	4104 (28.1)	90.01	** < 0.0001**
Chills	3964 (25.3)	81.63	** < 0.0001**
Diarrhea	2580 (17.7)	80.12	0.622
Dyspnea/chest pain	2497 (17.1)	84.34	** < 0.0001**
Abdominal pain	1484 (10.2)	80.20	0.784
Conjunctivitis	1117 (7.7)	83.89	**0.003**
Anosmia	1108 (7.6)	95.31	** < 0.0001**
Ageusia	808 (5.5)	95.30	** < 0.0001**
Vomit	615 (4.2)	81.63	0.497
Anosmia/ageusia	744 (5.1)	96.10	** < 0.0001**
Fever/myalgia or arthralgia/headache/cough	1285 (8.8)	92.61	** < 0.0001**
Dyspnea/throat pain/ runny nose/chills	474 (3.3)	85.65	**0.004**
Diarrhea/abdominal pain/vomit	169 (1.2)	80.47	1.000

*p-values for *x*^2^ test seroprevalence within symptoms.

^a^Total of seropositive cases = 11,577; It should be noted that some categories could be counted more than once. P-values in bold show which are equal to or less than 0.05.

### Natural seroconversion in unvaccinated people

**Figure 2:**
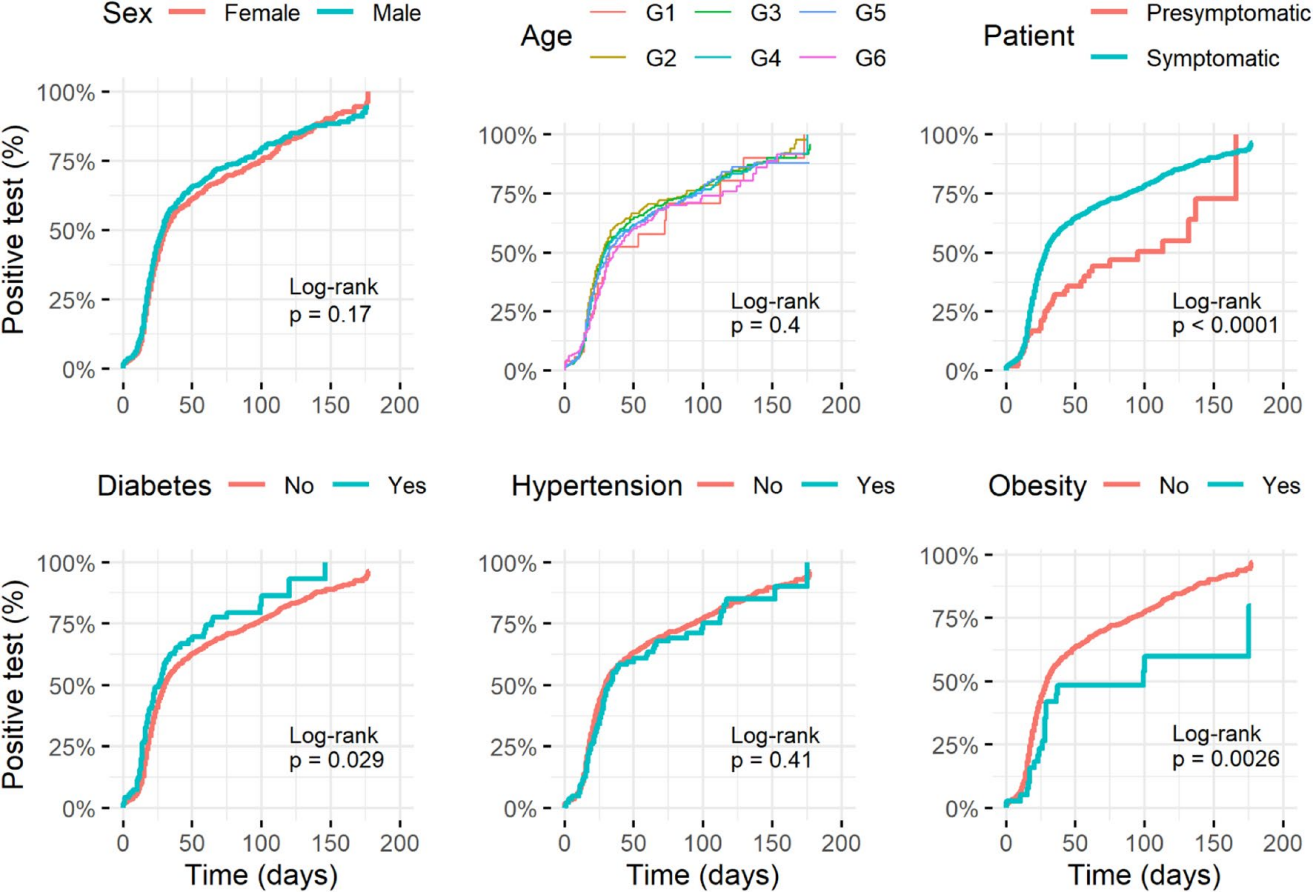
Seroconversion dynamic according to clinical characteristics in patients with COVID-19. Kaplan–Meier curves from the time COVID-19 was diagnosed by PCR test to a positive anti-SARS-CoV-2 antibodies result. The curves present the cumulative incidence of positive antibodies test in 1655 individuals diagnosed with COVID-19 and have a specific characteristic (blue) versus those who did not have the characteristic of interest (red) in time. Only sex and age Kaplan–Meier curves come from crude analysis. Age was included in models by decades: G1: < 20y, G2 = 20–29y, G3 = 30–39y, G4 = 40–49y, G5 = 50–59y, G6: ≥ 60y. Statistical details are in Supplementary Table S5.

We analyzed the seroconversion by time to event analysis in people previously diagnosed with COVID-19 (characteristics of the seroconverted cohort are in Supplementary Table S4). Hazard ratios (HR) for each characteristic, calculated by Cox proportional hazards models, are presented in Supplementary Table S5. Crude models showed that sex and age did not significantly impact the seroconversion (Fig. 2 and Supplementary Table S5); however, due to our previous observations (Table 1), we adjusted models for age and sex to evaluate each characteristic versus time (number of days since positive PCR test).

We found that diabetic people slightly increased seroconversion (HR = 1.39, 95% CI = 1.09–1.78) and obesity reduced it (HR = 0.53, 95%CI = 0.34–0.82) than healthy people, respectively. However, hypertension was not significant (HR = 0.91, 95% CI = 0.76–1.20). Further analyses revealed that symptomatic people have higher seroconversion than presymptomatic individuals (HR = 2.16, 95% CI = 1.58–2.94) (Fig. 2).

**Figure 3:**
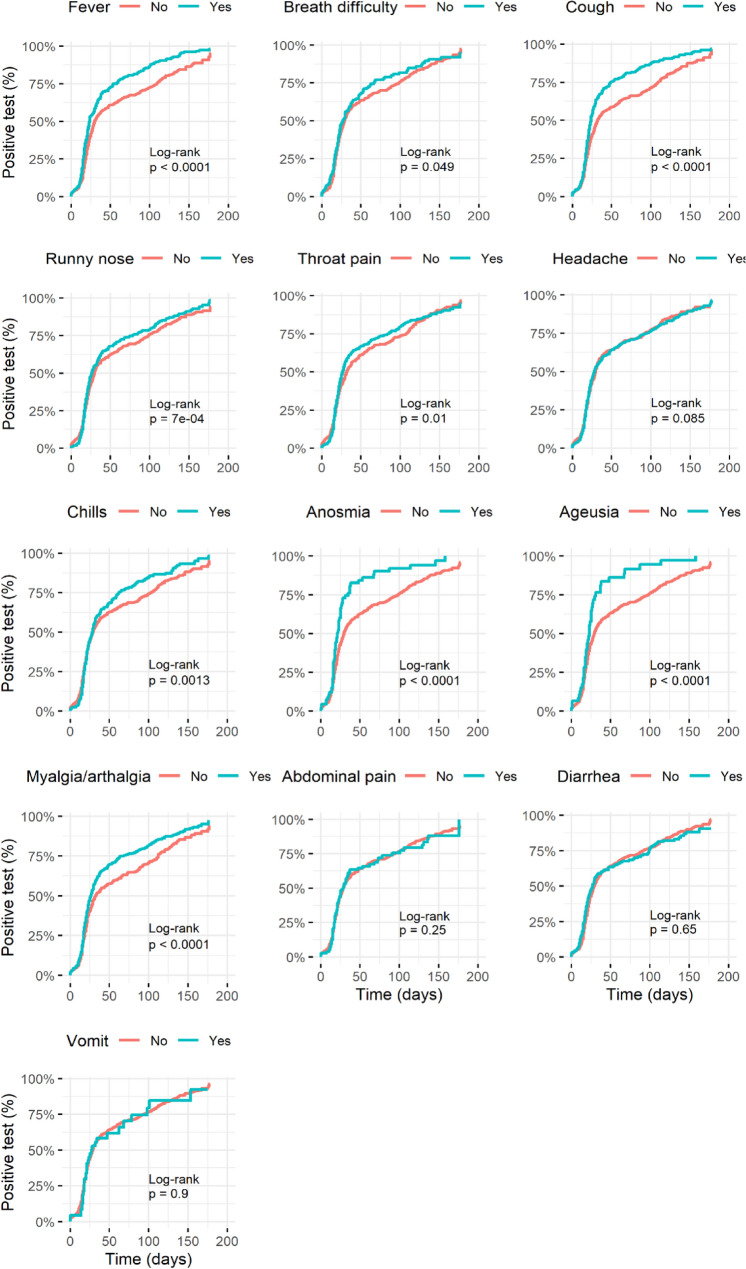
Seroconversion dynamic according to symptoms related to COVID-19. Kaplan–Meier curves from the time COVID-19 was diagnosed by PCR test to a positive anti-SARS-CoV-2 antibodies result. The curves present the cumulative incidence of positive antibodies test in 1100 individuals diagnosed with COVID-19 that had each symptom (blue) versus those who did not have the symptom of interest (red) in time. Presymptomatic individuals were excluded from this analysis. Statistical details are in Supplementary Table S5.

Individual symptoms revealed an intricate pattern, such as fever (HR = 1.44, 95%CI = 1.24–1.68) and myalgia/arthralgia (HR = 1.31, 95%CI = 1.14–1.51) significantly increased the frequency of seroconversion in less time than people that do not experience those symptoms. In contrast, respiratory symptoms such as breath difficulty (HR = 1.13, 95%CI = 0.94–1.36), throat pain (HR = 1.12, 95%CI = 0.98–1.29), and runny nose (HR = 1.12, 95%CI = 0.97–1.30) were not significantly related to increasing seroconversion frequency except the cough (HR = 1.47, 95%CI = 1.27–1.70) (Fig. 3).

Moreover, anosmia (HR = 1.72, 95%CI = 1.32–2.24) and ageusia (HR = 1.73, 95%CI = 1.27–2.36) significantly increased seroconversion frequency in less time than people that do not experience it. On the other hand, gastric symptoms such as diarrhea (HR = 1.02, 95%CI = 0.86–1.22), abdominal pain (HR = 1.03, 95%CI = 0.81–1.30), and vomit (HR = 1.03, 95%CI = 0.72–1.46) were not significantly related to increasing seroconversion frequency (Fig. 3).

Moreover, we evaluated the seroconversion in common symptomatic groups; we found that these groups had similar patterns of the individual symptoms included (Supplementary Table S5 and Supplementary Fig. S4).

## Discussion

This report aimed to study the prevalence and seroconversion of natural anti-SARS-CoV-2 antibodies to help estimate the burden of infections during the first two waves of the pandemic in Mexico. This study comprises the July to December of 2020, a period before vaccination began on December 24th, 2020; therefore, these results belong to the unvaccinated population.

We found a seroprevalence of 32.8% in the population studied; similar results to the IgG prevalence reported in outpatients tested in public health clinics at the end of December 2020^12^. These results suggest that around one-third of people tested in public and private Mexican health services were infected with SARS-CoV-2. However, most of the population remained susceptible at that time. Moreover, studies from other Latin-American countries report cumulative seroprevalence ranged from 20 to 41% in that period evidencing the high transmission of the virus in the community^19,20^.

Also, the seroprevalence varied across the country; however, it tends to increase in Northern states like Chihuahua, Coahuila, Sinaloa, and Sonora. The only exception was Tabasco, located in the Southeast region of Mexico; this pattern could be due to the differences in confinement measurements implemented by each state, disease onset, sampling method, and temporality of the pandemic in each region^10,11,13^.

Furthermore, we identified that young people aged 30 to 39 years had a higher prevalence of antibodies (38.5%); this could be due to the age distribution of the population in the country, and the majority could be economically active, which might have high occupational exposure. For example, we found that jobs like delivery people, logistics, transporting, and informal traders had higher seropositivity similar to previous reports^21–23^.

Recent works showed that people with previous comorbidities had more risk to develop severe COVID-19^24,25^ that could be related to more robust antibody response^26,27^; then, we analyzed the impact of chronic diseases in seroconversion.

We found a significant increase in the seroconversion frequency in diabetic people than healthy people. Other factors not evaluated, such as glycemic levels at the moment test; if the patient has treatment or not, their adherence to it, and time elapsed since disease diagnosis, the above could confuse the effect observed.

Furthermore, we found that obese people significantly had less seroconversion frequency than healthy people; this could be due to impair immune function^28^, as observed against other respiratory viruses such as flu^29,30^ and recently SARS-CoV-2^31^. However, due to obesity being self-reported, we could not accurately identify obese people or stratified, then these results should be taken with caution.

On the other hand, symptomatic people significantly increased their HR for seroconversion than presymptomatic. Recent works show that adaptive immunity is strongly activated in presymptomatic people; however, T cell and antibody response may differ^6,32,33^ that could explain low seroconversion frequency in time observed in presymptomatic people.

Furthermore, self-reported symptoms could help identify COVID-19 cases in regions with limited PCR or serological tests access. In this regard, we assess the association of self-reported symptoms with seropositivity. Some booster seroconversion, despite most symptoms showing a statistically significant association with seropositivity. For example, fever and myalgia are highly associated with the immune response, not exclusively for infections. Also, anosmia and ageusia boosted natural seroconversion consistent with previous reports^34^. While headache and sore throat do not significantly impact natural seroconversion; similarly, allergy-like and gastric-related symptoms (Supplementary Table 4).

Moreover, the presence of some mixed symptoms such as anosmia and ageusia were more specific to COVID-19 (715/744 = 96.1%; p = 0.0001) and also increased hazard ratio for seroconversion. Similarly, other group of symptoms, such as fever, myalgia/arthralgia, and cough (1190/1285 = 92.6%; p = 0.0001) that also increased hazard ratio for seroconversion than single symptoms (Supplementary Table 4). However, gastric symptoms like diarrhea, abdominal pain, and vomit previously described in cluster symptoms in COVID-19 patients^17^ are unrelated to increasing seroconversion. These observations are concordant with previous reports that showed that symptoms like anosmia/ageusia, dyspnea, fever, and their combination are strongly associated with SARS-CoV-2 seropositivity^23,35^. The above is of particular interest for empirical COVID-19 diagnosis in areas with insufficient testing and to predict seroconversion in people due to their intrinsic characteristics and the symptoms developed during infection, which could be crucial for back to work algorithms and other contentions actions against COVID-19.

However, it is necessary to consider that outbreaks of different SARS-CoV-2 variants could promote specific symptoms^36,37^; then, continuous surveillance would help to improve our knowledge about natural immunity responses against the virus for a comprehensive understanding of the clinical manifestations of COVID-19.

Our study has several strengths, highlighting the large sample size used to estimate the prevalence of anti-SARS-CoV-2 antibodies in most country regions; with this, we could analyze individuals from all states of Mexico during the first months of the pandemic before vaccination. The above is particularly important because it allowed us to better characterizer the community settings' clinical spectrum of symptoms experienced by COVID-19 and the influence of seroconversion-related factors.

Some limitations of this study included that information of the registry analyzed did not come from a population-based sampling, which makes an underrepresentation of population groups; also, we did not know the duration and severity of symptoms experienced due to their self-reported nature in people diagnosed with COVID-19.

Additionally, analysis of clinical and lifestyle factors covers 13 of 32 states; however, they have 65.5% of the population-wide in five regions (Northwest, Northeast, Center, Occident, and South). Some of them, such as the southern region, have lower representation due to fewer cases with complete clinical and demographic information.

Because we studied outpatients in a community setting, it was impossible to follow up with each to collect serial samples for antibodies testing. Instead, we made a cohort-based on a convenient sampling of people confirmed with COVID-19 by PCR test and serial antibodies tests till 180 days after PCR test for time to event analysis. Then, the number of people analyzed was reduced.

Nevertheless, our study provides an overview of SARS-CoV-2 seroprevalence in the unvaccinated Mexican population that contributes to understanding the Americas' pandemic impact. Also, these findings allow us to identify significant characteristics and symptoms related to natural seroconversion, especially those that boost this response in less time; this could be considered for clinical management of infected people and vaccination.

Together, these results contribute to characterizing the natural immunity induced by SARS-CoV-2 and comparing it with the antibody-mediated immunity induced by vaccination, helping us design effective vaccination schemes against SARS-CoV-2, prioritizing risk groups to fine-tune algorithms for back to work based on serological testing.

## Supplementary Information

Supplementary Information.

## Data Availability

All relevant data concerning this work are published in this article and the supplementary material.
