# Supplementary material for: Seroconversion dynamic and SARS-CoV-2 seropositivity in unvaccinated population during the first and second outbreaks in Mexico
Source: Sci Rep. 2022 Mar 28;12:5241. doi: 10.1038/s41598-022-09395-3 (PMC8960100; doi:10.1038/s41598-022-09395-3)
Supplement: Supplementary file 1 — Supplementary Information. [file 41598_2022_9395_MOESM1_ESM.docx]

**Seroconversion dynamic and SARS-CoV-2 seropositivity in unvaccinated population during the first and second outbreaks in Mexico**

**(Supplementary information)**

Miguel A. Fernández-Rojas^1^, Marco A. Luna–Ruiz Esparza^1^, Abraham Campos Romero^1^, Diana Y. Calva-Espinosa^2^, José L. Moreno-Camacho^2,3^, Fela Mendlovic^4,5^, Tanya Plett-Torres^6^ and Jonathan Alcántar-Fernández^1^ (*****)

**Affiliations:**

^1^Innovation and Research Department, Salud Digna, Culiacan, 80000, Sinaloa, Mexico.

^2^Clinical Laboratory Department, Salud Digna, Culiacan, 80000, Sinaloa, Mexico.

^3^National Reference Center “Culiacan”, Salud Digna, Culiacan,  80300, Sinaloa, Mexico.

^4^Departamento de Microbiología y Parasitología, Facultad de Medicina, Universidad Nacional Autónoma de México, Ciudad de Mexico, Mexico.

^5^Facultad de Ciencias de la Salud, Universidad Anáhuac México Norte, Huixquilucan, Estado de Mexico, Mexico.

^6^Plan de Estudios Combinados en Medicina, Facultad de Medicina, Universidad Nacional Autónoma de México, Ciudad de Mexico, Mexico.

**Corresponding author**

Jonathan Alcántar-Fernández, Ph.D. Innovation and Research Department, Salud Digna A.C., Francisco Villa 113 sur, 80000, Culiacan Sinaloa, Mexico. Phone: (55)6677137521. Email: [jonathan.alcantar@salud-digna.org](mailto:jonathan.alcantar@salud-digna.org)

**Table S1. Geographical seroprevalence to anti-SARS-CoV-2 antibodies in Mexico.**

| **State** | **Number of people** | **People with antibodies** | **ASR^a^** | **95% CI** | ***p-valu*e** |
| --- | --- | --- | --- | --- | --- |
| National | 522,690 | 186,663 | 32.8 | 32.1-33.5 | ref |
| Aguascalientes | 3,212 | 743 | 22.7 | 19.3-26.1 | <0.01 |
| Baja California | 26,484 | 9,410 | 34.0 | 32.8-35.3 | n.s |
| Baja California Sur | 1,017 | 354 | 38.5 | 29.1-48.0 | n.s |
| Campeche | 1,098 | 320 | 27.3 | 22.7-32.0 | <0.05 |
| Chiapas | 3,943 | 1,007 | 26.4 | 21.9-30.9 | <0.01 |
| Chihuahua | 18,814 | 8,037 | 41.2 | 39.6-42.8 | <0.01 |
| Ciudad de México | 74,345 | 27,248 | 35.9 | 35.1-36.7 | <0.01 |
| Coahuila | 8,924 | 3,618 | 38.8 | 36.6-40.9 | <0.01 |
| Colima | 2,914 | 480 | 18.1 | 14.5-21.6 | <0.01 |
| Durango | 4,930 | 1,761 | 34.2 | 30.9-37.5 | n.s |
| Estado de México | 98,813 | 35,894 | 34.9 | 34.2-35.6 | <0.01 |
| Guanajuato | 24,403 | 6,703 | 28.2 | 26.8-29.6 | <0.01 |
| Guerrero | 1,829 | 606 | 32.1 | 27.8-36.5 | n.s |
| Hidalgo | 4,513 | 1,570 | 35.2 | 31.8-38.6 | n.s |
| Jalisco | 34,796 | 11,158 | 30.8 | 29.7-31.8 | <0.01 |
| Michoacán | 12,803 | 3,738 | 28.6 | 27.0-30.3 | <0.01 |
| Morelos | 2,420 | 640 | 26.7 | 23.0-30.4 | <0.01 |
| Nayarit | 2,532 | 614 | 23.7 | 19.1-28.3 | <0.01 |
| Nuevo León | 35,283 | 14,773 | 40.7 | 39.4-42.0 | <0.01 |
| Oaxaca | 1,510 | 511 | 32.6 | 25.7-39.5 | n.s |
| Puebla | 18,983 | 5,329 | 27.4 | 25.9-29.0 | <0.01 |
| Querétaro | 17,653 | 4,376 | 23.9 | 22.6-25.2 | <0.01 |
| Quintana Roo | 4,551 | 1,798 | 35.7 | 32.4-39.0 | n.s |
| San Luis Potosí | 4,461 | 1,457 | 30.5 | 27.3-33.6 | n.s |
| Sinaloa | 60,111 | 24,959 | 40.9 | 39.9-41.9 | <0.01 |
| Sonora | 14,565 | 5,828 | 39.5 | 37.4-41.5 | <0.01 |
| Tabasco | 3,889 | 1,891 | 51.3 | 46.0-56.5 | <0.01 |
| Tamaulipas | 6,923 | 2,679 | 37.3 | 34.6-40.0 | <0.01 |
| Tlaxcala | 1,796 | 680 | 33.7 | 29.8-37.7 | n.s |
| Veracruz | 21,679 | 7,307 | 32.3 | 30.9-33.7 | n.s |
| Yucatán | 1,249 | 450 | 37.6 | 27.4-47.8 | n.s |
| Zacatecas | 2,247 | 724 | 29.1 | 25.0-33.2 | n.s |

Abbreviatures: ^a^ASR= age and sex standardized rate per 100 inhabitants.; ref= reference category; n.s= not significant; 95% CI: 95% confidence interval.

Note: Standardization was calculated with the direct method, using the standard world population as a reference: World (WHO 2000-2025).

**Table S2. Occupations of people with anti-SARS-CoV-2 antibodies previously diagnosed with COVID-19**

| **Occupations**  **(n= 14,592)** | **Number of people (%)** | **Prevalence of antibodies (%)** | ***OR* (95% CI)** | ***p-value*** |
| --- | --- | --- | --- | --- |
| Healthcare worker | 607 | 76.0 | ref | --- |
| Customer service | 907 | 80.2 | 1.3 (1.0 – 1.7) | 0.046 |
| Delivery person | 87 | 87.4 | 2.2 (1.1 – 4.2) | 0.019 |
| Farmer/Rancher | 125 | 76.0 | 1.0 (0.6 - 1.6) | 0.981 |
| Informal trader/Salesman | 779 | 81.5 | 1.4 (1.1 – 1.8) | 0.010 |
| Logistic and Transport | 383 | 83.3 | 1.6 (1.2 – 2.2) | 0.006 |
| Non-specified | 4,582 | 80.8 | 1.3 (1.1 – 1.6) | 0.005 |
| Office worker | 4,035 | 78.9 | 1.2 (1.0 – 1.5) | 0.094 |
| Public Servant | 486 | 78.6 | 1.2 (0.9 – 1.6) | 0.283 |
| School staff | 916 | 76.6 | 1.0 (0.8 – 1.3) | 0.827 |
| Student | 904 | 76.0 | 1.1 (0.8 – 1.3) | 0.978 |
| Unemployed | 713 | 78.0 | 1.1 (0.9 – 1.5) | 0.374 |

Abbreviatures: OR= odd ratio; ref=reference category. Occupations were included as categorical variables in the model.

**Table S3. Comorbidities of 14,592 people with SARS-CoV-2 antibodies previously diagnosed with COVID-19.**

| **Comorbidities** | **Number of people (%)** | **Prevalence of antibodies (%)** | **Adjusted *OR***  **(95% CI)** | ***p-valu*e** |
| --- | --- | --- | --- | --- |
| Smoking | 2,367 (16.2) | 76.7 | 0.8 (0.7 – 0.9) | 0.0001 |
| Hypertension | 1,033 (7.1) | 79.9 | 0.9 (0.8 – 1.1) | 0.312 |
| Diabetes | 849 (5.8) | 83.0 | 1.3 (1.0 – 1.5) | 0.026 |
| Obesity | 354 (2.4) | 79.4 | 1.0 (0.7 – 1.3) | 0.702 |
| Hyper/ Hypothyroidism | 198 (1.4) | 78.8 | 1.0 (0.7 – 1.5) | 0.826 |
| Asthma | 162 (1.1) | 78.4 | 1.0 (0.7 – 1.5) | 0.989 |
| Arthritis | 81 (0.6) | 74.1 | 0.7 (0.4 – 1.2) | 0.219 |
| Cardiac disease | 96 (0.7) | 78.1 | 0.9 (0.5 – 1.4) | 0.533 |
| Cancer | 60 (0.4) | 78.3 | 0.9 (0.5 – 1.7) | 0.716 |
| Renal insufficiency | 30 (0.2) | 76.7 | 0.8 (0.4 – 1.9) | 0.647 |
| HIV | 23 (0.2) | 95.7 | 5.6 (0.8 – 41.8) | 0.092 |
| Pulmonary hypertension | 19 (0.1) | 94.7 | 4.9 (0.7 – 37.2) | 0.122 |
| COPD | 18 (0.1) | 77.8 | 0.9 (0.3 – 2.7) | 0.799 |
| Lupus | 20 (0.1) | 65.0 | 0.5 (0.2 – 1.3) | 0.172 |
| Hepatic insufficiency | 8 (0.1) | 75.0 | 0.7 (0.1 – 3.6) | 0.697 |

Notes: OR was adjusted by age and sex; the age was included in the model as a categorical variable as <20, 20-29, 30-39,40-49,50-59, and >60 years. Similarly, each comorbidity listed in the table was included as categorical variables (Yes/No) in the model; the reference category was negative cases in each category (comorbidity). Total of seropositive cases=11,577; It should be noted that some categories could be counted more than once.

**Table S4. Characteristics of the seroconversion cohort**

| **Characteristic** | **Number of people** | **Frequency (%)** |
| --- | --- | --- |
| **Sex** |  |  |
| Female | 914 | 55.23 |
| Male | 741 | 44.77 |
| **Age (years)** |  |  |
| <20 | 50 | 3.02 |
| 20-29 | 374 | 22.60 |
| 30-39 | 436 | 26.34 |
| 40-49 | 360 | 21.75 |
| 50-59 | 261 | 15.77 |
| ≥60 | 174 | 10.51 |
| **Diabetes** |  |  |
| Yes | 94 | 5.68 |
| No | 1,561 | 94.32 |
| **Hypertension** |  |  |
| Yes | 126 | 7.61 |
| No | 1,529 | 92.39 |
| **Obesity** |  |  |
| Yes | 38 | 2.30 |
| No | 1,617 | 97.70 |
| **Type of patient** |  |  |
| Symptomatic | 1,100 | 66.47 |
| Presymptomatic | 555 | 33.53 |
| **Cough*** |  |  |
| Yes | 343 | 31.18 |
| No | 757 | 68.82 |
| **Fever*** |  |  |
| Yes | 296 | 26.90 |
| No | 804 | 73.10 |
| **Dyspnea*** |  |  |
| Yes | 171 | 15.55 |
| No | 929 | 84.45 |
| **Throat pain*** |  |  |
| Yes | 575 | 52.27 |
| No | 525 | 47.73 |
| **Myalgia/Arthralgia*** |  |  |
| Yes | 573 | 52.09 |
| No | 527 | 47.91 |
| **Runny nose*** |  |  |
| Yes | 341 | 31.00 |
| No | 759 | 69.00 |
| **Headache*** |  |  |
| Yes | 690 | 62.73 |
| No | 410 | 37.27 |
| **Chills*** |  |  |
| Yes | 241 | 21.91 |
| No | 859 | 78.09 |
| **Anosmia*** |  |  |
| Yes | 66 | 6.00 |
| No | 1034 | 94.00 |
| **Ageusia*** |  |  |
| Yes | 46 | 41.82 |
| No | 1,054 | 95.82 |
| **Abdominal pain*** |  |  |
| Yes | 103 | 9.36 |
| No | 997 | 90.64 |
| **Diarrhea*** |  |  |
| Yes | 227 | 20.64 |
| No | 873 | 79.36 |
| **Vomit*** |  |  |
| Yes | 47 | 4.27 |
| No | 1,053 | 95.73 |

Notes: (*) presymptomatic patients were excluded

**Table S5. Hazard ratio (HR) analysis for seroconversion**

| **Characteristic** | **Crude model** | | **Adjusted model** | | |
| --- | --- | --- | --- | --- | --- |
|  | **HR** | **95% CI** | **HR** | **95% CI** | **p value** |
| Sex |  |  |  |  |  |
| Male | 1.08 | 0.97-1.22 | - | - | - |
| Female | 1 | - | - | - | - |
| Age group (years) |  |  |  |  |  |
| <20 | 1 | - | - | - | - |
| 20-29 | 1.24 | 0.85-1.81 | - | - | - |
| 30-39 | 1.14 | 0.79-1.66 | - | - | - |
| 40-49 | 1.12 | 0.77-1.63 | - | - | - |
| 50-59 | 1.09 | 0.74-1.61 | - | - | - |
| ≥60 | 0.98 | 0.66-1.46 | - | - | - |
| **Chronic diseases** |  |  |  |  |  |
| Diabetes | 1.30 | 1.03-1.65 | **1.39** | **1.09-1.78** | **0.008** |
| Obesity | 0.51 | 0.33-0.80 | **0.53** | **0.34-0.82** | **0.005** |
| Hypertension | 0.91 | 0.73-1.14 | 0.95 | 0.76-1.20 | 0.67 |
| **Presence of symptoms** |  |  |  |  |  |
| Symptomatic | 2.13 | 1.57-2.90 | **2.16** | **1.58-2.94** | **<0.0001** |
| **Symptoms** |  |  |  |  |  |
| Fever | 1.45 | 1.25-1.68 | **1.44** | **1.24-1.68** | **<0.0001** |
| Cough | 1.47 | 1.27-1.70 | **1.47** | **1.27-1.70** | **<0.0001** |
| Myalgias and arthralgias | 1.30 | 1.13-1.50 | **1.31** | **1.14-1.51** | **<0.0001** |
| Dyspnea | 1.12 | 0.93-1.34 | 1.13 | 0.94-1.36 | 0.20 |
| Throat pain | 1.11 | 0.97-1.28 | 1.12 | 0.98-1.29 | 0.11 |
| Chills | 1.16 | 0.99-1.37 | 1.16 | 0.99-1.34 | 0.07 |
| Runny nose | 1.14 | 0.98-1.32 | 1.12 | 0.97-1.30 | 0.14 |
| Anosmia | 1.70 | 1.31-2.21 | **1.72** | **1.32-2.24** | **<0.0001** |
| Ageusia | 1.73 | 1.27-2.36 | **1.73** | **1.27-2.36** | **0.0005** |
| Headache | 1.00 | 0.86-1.15 | 0.98 | 0.85-1.14 | 0.83 |
| Diarrhea | 1.02 | 0.82-1.21 | 1.02 | 0.86-1.22 | 0.81 |
| Abdominal pain | 1.00 | 0.79-1.26 | 1.03 | 0.81-1.30 | 0.84 |
| Vomit | 0.99 | 0.70-1.40 | 1.03 | 0.72-1.46 | 0.88 |
| **Symptoms groups** |  |  |  |  |  |
| Anosmia/ageusia | 1.71 | 1.25-2.34 | **1.70** | **1.24-2.34** | **0.001** |
| Fever myalgia, arthralgia, headache, and cough | 1.79 | 1.42-2.24 | **1.75** | **1.39-2.20** | **<0.0001** |
| Chills, runny nose, and throat pain | 0.98 | 0.64-1.50 | 0.97 | 0.63-1.50 | 0.89 |
| Diarrhea, vomit, and abdominal pain | 1.09 | 0.56-2.04 | 1.14 | 0.61-2.12 | 0.69 |

Notes: For crude and adjusted models, the control groups for cox regression were individuals that did not have the characteristic of interest. We exclude presymptomatic people for symptoms and symptoms groups; then, we analyze 1,100 individuals. Models were adjusted for sex and age in groups of decades. Results in bold indicate characteristics statistically significant to seroconversion at p<0.05 statistical threshold.


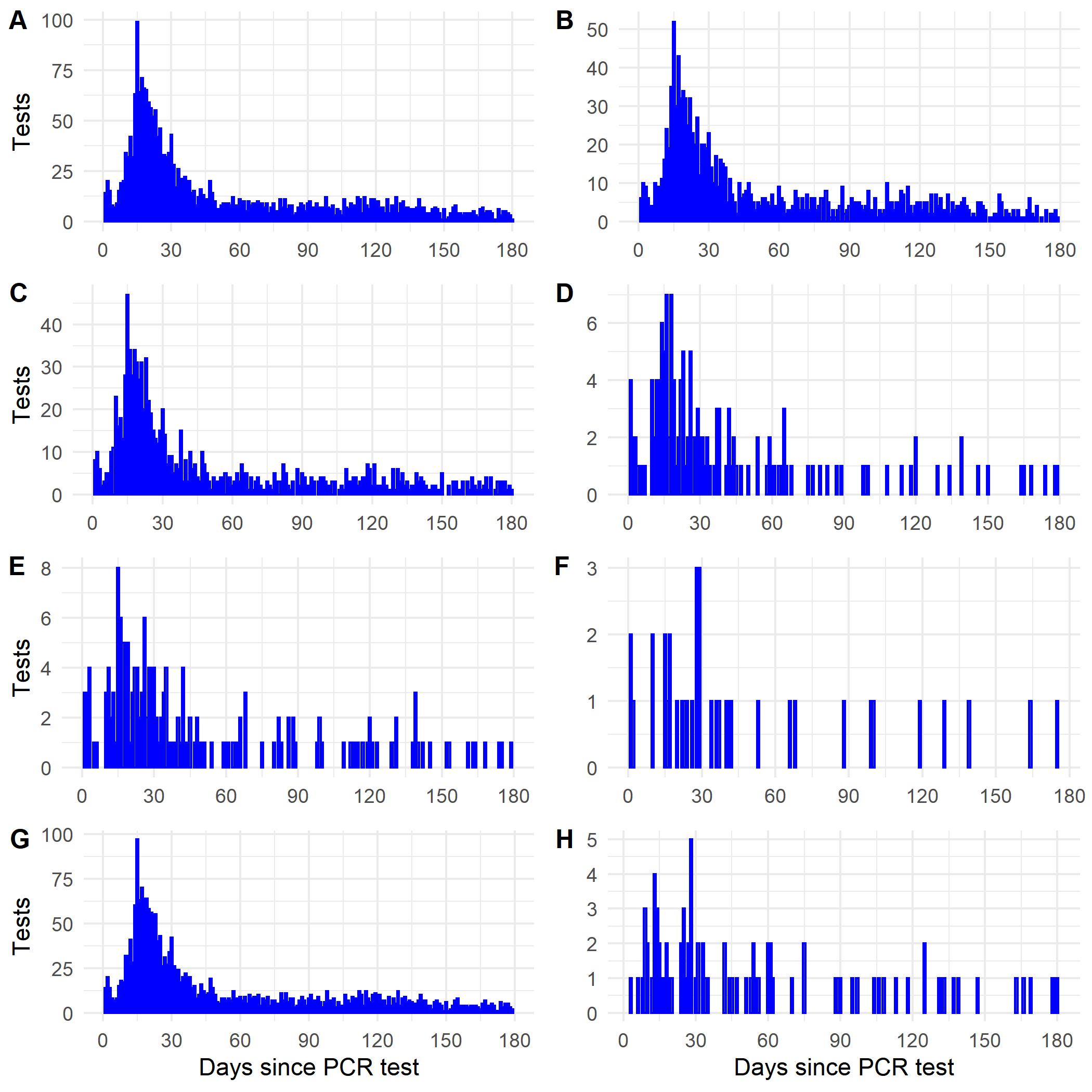


**Fig. S1 Distribution of serial serological tests overtime in people belonging to the seroconversion cohort according to clinical characteristics.**

Histograms shows the frequency of serial serological tests in the cohort for seroconversion analysis in 1,655 individuals. **A)** Overall, **B)** Female, **C)** Male, **D)** Diabetes, **E)** Hypertension, **F)** Obesity, **G)** Symptomatic people, and **H)** Presymptomatic people.


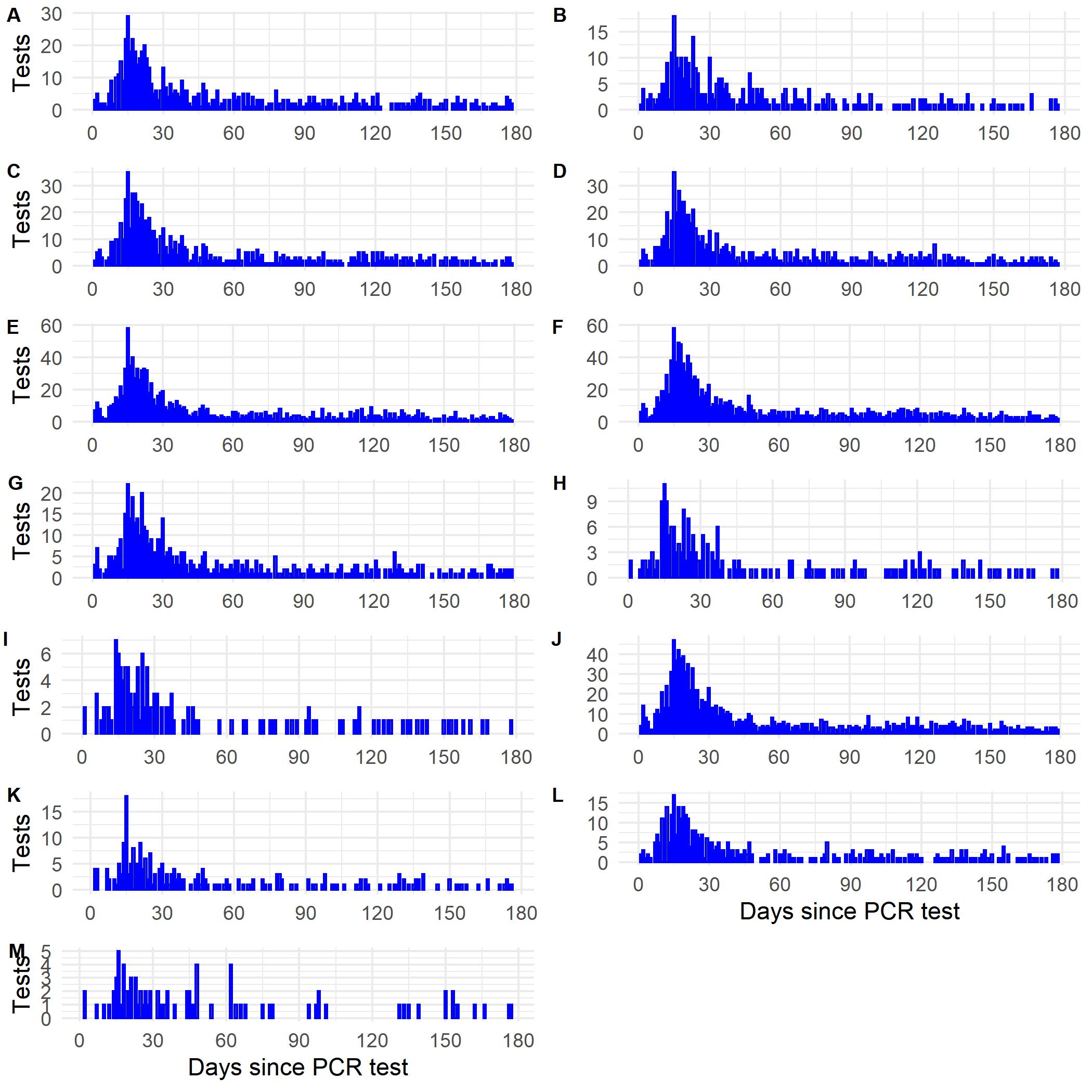


**Fig. S2 Distribution of serial serological tests overtime in symptomatic people belonging to the seroconversion cohort according to their symptoms.**

Histograms shows the frequency of serial serological tests in the cohort for seroconversion analysis in 1,100 symptomatic people **A)** Fever, **B)** Dyspnea, **C)** Cough, **D)** Runny nose, **E)** Throat pain, **F)** Headache, **G)** Chills, **H)** Anosmia, **I)** Ageusia, **J)** Myalgias and arthralgias, **K)** Abdominal pain, **L)** Diarrhea, and **M)** Vomit.


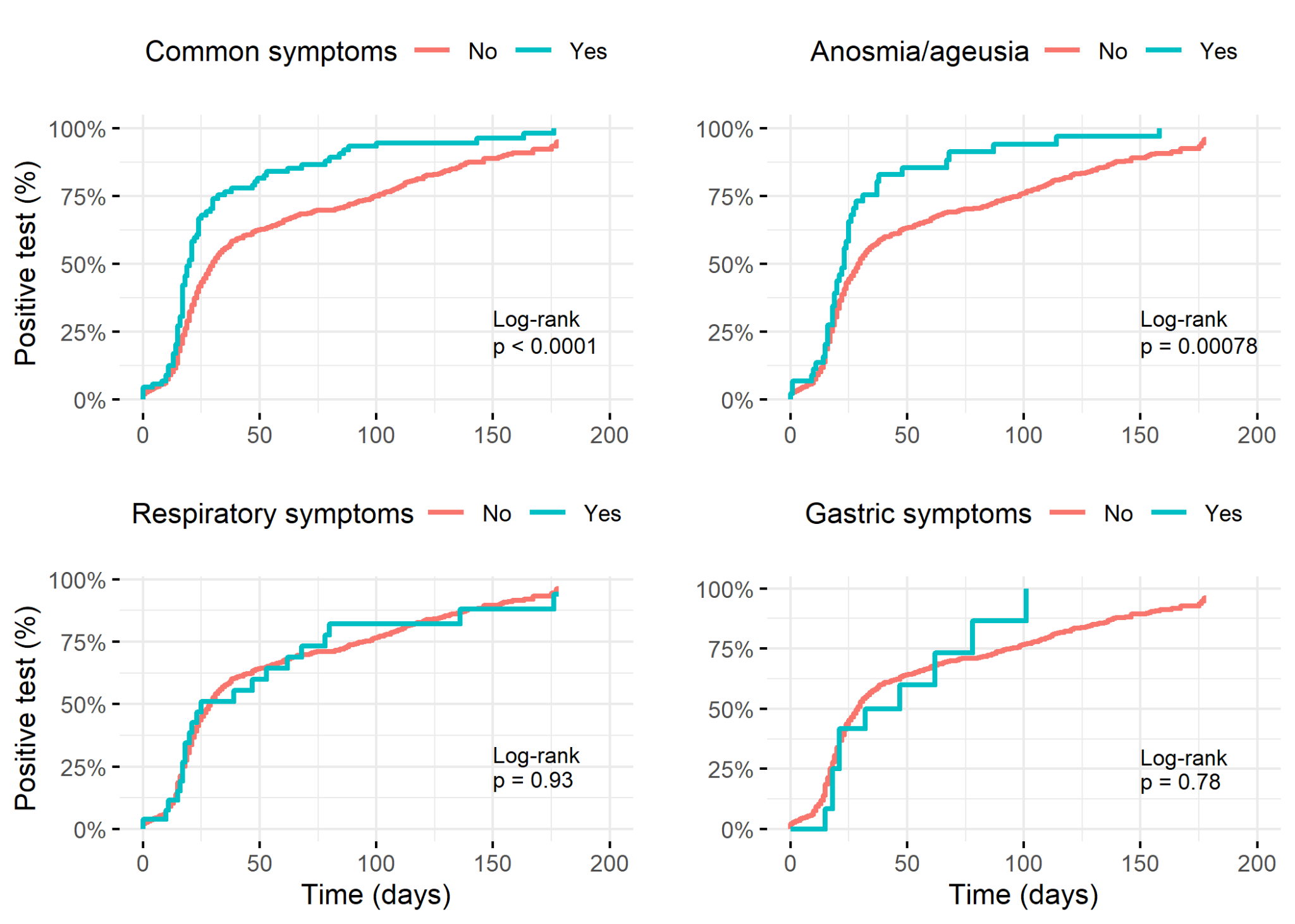


**Fig. S3 Seroconversion dynamic according to groups of symptoms related to COVID-19**

Kaplan–Meier curves from the time COVID-19 was diagnosed by PCR test to a positive anti-SARS-CoV-2 antibodies result. The curves present the cumulative incidence of positive antibodies test in 1,100 individuals diagnosed with COVID-19 classified in groups according to the self-reported symptoms reported at PCR test (blue) versus those who did not report any symptoms in the group of interest (red) in time. The groups of symptoms were the following: common=fever, myalgia/arthralgia, headache, and cough; anosmia/ageusia=anosmia and ageusia; respiratory symptoms=chills, runny nose, and throat pain; gastric symptoms=diarrhea, abdominal pain, and vomit. Presymptomatic people were excluded from the analysis. Statistical details are in Supplementary Table S5.
